# The Association between Early Changes in Neutrophil-Lymphocyte Ratio and Survival in Patients Treated with Immunotherapy

**DOI:** 10.3390/jcm11154523

**Published:** 2022-08-03

**Authors:** Deniz Can Guven, Taha Koray Sahin, Enes Erul, Ibrahim Yahya Cakir, Enes Ucgul, Hasan Cagri Yildirim, Oktay Halit Aktepe, Mustafa Erman, Saadettin Kilickap, Sercan Aksoy, Suayib Yalcin

**Affiliations:** 1 Department of Medical Oncology, Cancer Institute, Hacettepe University, 06100 Ankara, Turkey; hasan-cagri@windowslive.com (H.C.Y.); droktayaktepe@hotmail.com (O.H.A.); ermanm1968@gmail.com (M.E.); skilickap@yahoo.com (S.K.); saksoy@hacettepe.edu.tr (S.A.); suayibyalcin@gmail.com (S.Y.); 2 Department of Internal Medicine, Faculty of Medicine, Hacettepe University, 06100 Ankara, Turkey; takorsah@gmail.com (T.K.S.); eneserul@hacettepe.edu.tr (E.E.); ibrahimyahyacakir@gmail.com (I.Y.C.); enes-ucgul@hotmail.com (E.U.); 3 Department of Medical Oncology, Faculty of Medicine, Istinye University, 34396 Istanbul, Turkey

**Keywords:** biomarker, cancer, Charlson Comorbidity Index, immunotherapy, neutrophil–lymphocyte ratio, NLR2-CEL

## Abstract

Dynamic changes in the blood-based biomarkers could be used as a prognostic biomarker in patients treated with immune checkpoint inhibitors (ICIs), although the data are limited. We evaluated the association between the neutrophil–lymphocyte ratio (NLR) and early NLR changes with survival in ICI-treated patients. We retrospectively evaluated the data of 231 patients with advanced-stage cancer. We recorded baseline clinical characteristics, baseline NLR and fourth-week NLR changes, and survival data. A compound prognostic score, the NLR2-CEL score, was developed with the following parameters: baseline NLR (<5 vs. ≥5), ECOG status (0 vs. ≥1), Charlson Comorbidity Index (CCI, <9 vs. ≥9), LDH (N vs. ≥ULN), and fourth-week NLR change (10% or over NLR increase). In the multivariable analyses, higher NLR (HR: 1.743, *p* = 0.002), 10% or over NLR increase in the fourth week of treatment (HR: 1.807, *p* = 0.001), higher ECOG performance score (HR: 1.552, *p* = 0.006), higher LDH levels (HR: 1.454, *p* = 0.017), and higher CCI (HR: 1.400, *p* = 0.041) were associated with decreased OS. Compared to patients with the lowest scores, patients in the highest score group had significantly lower OS (HR: 7.967, 95% CI: 3.531–17.979, *p* < 0.001) and PFS. The composite score had moderate success for survival prediction, with an AUC of 0.702 (95% CI: 0.626–0.779, *p* < 0.001). We observed significantly lower survival in patients with higher baseline NLR values and increased NLR values under treatment.

## 1. Introduction

Immune checkpoint inhibitors (ICIs) added another dimension to cancer care with a unique mechanism of action and became the fifth pillar of oncologic treatments [1,2]. The ICIs demonstrated survival improvements in several tumors and entered treatment algorithms [3,4,5,6]. Long-term disease control and even a possible cure in the metastatic setting were reported in a significant portion of patients with relatively chemoresistant tumors, such as melanoma and renal cell carcinoma [7,8]. Furthermore, the use of ICI-based combination strategies improved the survival landmarks further, as evidenced by the unprecedented median overall survival of six years in metastatic melanoma [7] and a median survival of around four years in intermediate-high risk renal cell carcinoma [8] with nivolumab plus ipilimumab combination. 

Although deep and durable responses are possible with ICIs, the response rates are lower than 40% in most tumors, especially in the later lines of treatment, and toxicities, including class-specific immune-related adverse events, could be debilitating [9,10,11]. Additionally, most countries have limited access to immunotherapy due to the significant financial burden of immunotherapy [12]. These issues denote the need for biomarkers to aid in better patient selection for ICI use. 

Despite the stunning rate of ICI development and clinical trials, biomarker development has been relatively slow. Other than the tumor PD-L1 expression in the first-line ICI use in non-small cell lung cancer (NSCLC) and tumor agnostic use of tumor mutational burden (TMB) and microsatellite instability (MSI), no other biomarker has entered routine clinical use [13,14,15]. These biomarkers require a tissue section, and the TMB requires a complex platform [16]. Additionally, these biomarkers encompass a limited number of patients benefitting from ICIs [17]. Due to limitations with these biomarkers, there is a growing interest in blood-based biomarkers, which are readily available and could be serially evaluated in times of progression. 

While measuring the TMB, PD-L1, and lymphocyte immune profile is possible via the blood samples, simple biomarkers retrieved from complete blood count, such as the neutrophil–lymphocyte ratio (NLR), could be valuable for prognosis prediction also [18,19,20]. The higher levels of NLR were consistently associated with decreased survival with ICIs, possibly due to increased inflammatory pressure leading to immune exhaustion; however, the cutoffs for NLR and patient cohorts were very variable [21,22,23,24,25]. Additionally, the dynamic changes in NLR could reflect the changes in the immune machinery in response to ICIs and could present a minimally invasive way to monitor the host earlier in the treatment course [26,27,28]. Considering the instrumental role of adaptive immune system as a driver of ICI efficacy, the exploitation of NLR as a prognostic biomarker has a strong biologic rationale [29]. However, the studies evaluating the prognostic role of baseline NLR and early NLR changes are limited in ICI-treated patients. Furthermore, baseline NLR levels were previously used in the compound scoring systems incorporating several baseline laboratory and clinical parameters with the aim of treatment tailoring and prognosis estimations; however, none of the previously available compound prognostic scores was included the changes in NLR-levels in follow-up to the equations [30,31,32]. Therefore, we evaluated the association between NLR and early NLR changes with survival in ICI-treated patients. Additionally, we created an NLR-based compound prognostic score (NLR2-CEL score) and tested the efficacy of this score in a cohort of two institutions. 

## 2. Materials and Methods

### 2.1. Patient Population

**Figure 1:**
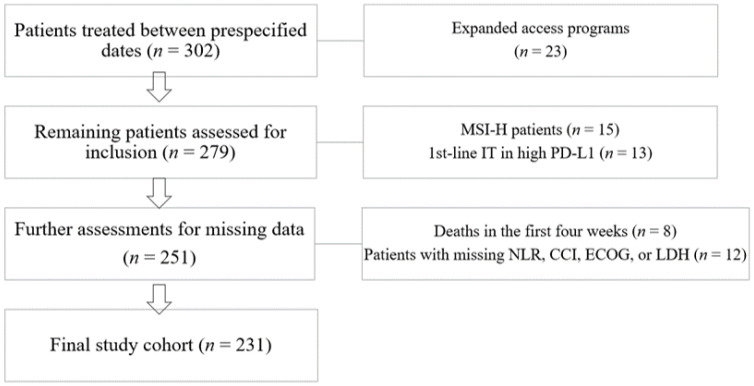
Flow diagram of patient selection process.

We retrospectively evaluated the adult (≥18 years of age) patients with advanced cancer treated with any ICI between January 2014 and August 2021 in two centers. We included all patients in the prespecified dates other than patients meeting prespecified exclusion criteria: (i) patients treated within the expanded access programs and clinical trials, (ii) biomarker selected patients, (iii) patients with missing laboratory or clinical data, and (iv) patients who died in the first four weeks of ICI treatment (Figure 1). We recorded the following variables from the patient files and hospital registry system: patient age, sex, Eastern Cooperative Oncology Group (ECOG) performance score, baseline height and weight, baseline and the fourth-week NLR, Charlson Comorbidity Index (CCI), immunotherapy line, metastatic sites at the start of ICIs, the best response to ICIs, and progression-free (PFS) and overall survival (OS). We recorded the type of ICI, the start and cessation dates of ICI, and the number of ICIs from the automated hospital treatment order system.

### 2.2. Statistical Analyses 

We expressed the baseline characteristics with medians and interquartile ranges (IQR) for continuous variables and frequencies and percentages for categorical variables. We used five as the cutoff value for baseline NLR and an NLR increase of 10% or greater (from baseline) for the fourth week of NLR change. We compared baseline characteristics of the prognostic groups with Chi-square and Kruskal–Wallis H tests. The OS was defined as the period from treatment initiation to the last follow-up and/or death, and PFS was defined as the period between treatment initiation to disease progression and/or death. We conducted survival analyses with Kaplan–Meier analyses and compared survival times between prognostic subgroups by the log-rank test. We conducted the multivariable analyses by the Cox regression analyses and calculated hazard ratios with 95% confidence intervals (CIs). The predictive performance of the NLR-based composite score for OS was assessed as receiver operating characteristic (ROCs) curves. Additionally, the performance of two previous compound scores, the Gustave Roussy Score (GRS) [30] and Royal Marsden Hospital Score (RMH) [31], were assessed by the ROC analyses. The statistical analyses were performed in SPSS, version 25.0 (IBM Inc., Armonk, NY, USA), and the ROC analyses were conducted with GraphPad Prism, version 8.0.0, for Windows (GraphPad Software, San Diego, CA, USA). A type-I error level of 5% (*p* < 0.05) was considered as the threshold limit for statistical significance.

### 2.3. Ethical Approval 

All procedures performed in studies involving human participants were under the ethical standards of the institutional and/or national research committee and with the 1964 Helsinki declaration and its later amendments or comparable ethical standards. The study was approved by the ethics committee of Istinye University.

## 3. Results

### 3.1. Baseline Characteristics

We included a total of 231 patients in the analyses (Figure 1). The median age of the cohort was 61 (IQR 51–67), and 67.1% of the patients were male. The most common diagnoses were RCC and melanoma. The 87% of the patients had 0 or 1 ECOG performance status. Most patients were treated in second or third lines (60.2%), and nivolumab was the most frequently used immunotherapy agent. The median CCI was 8, and the CCI high or low groups were defined according to this cutoff (Table 1). In the fourth-week evaluation, 97 patients (42%) had a 10% or higher increase in NLR levels compared to baseline values. The percentage of patients with 10% or higher increase in NLR levels was similar across different tumor types (melanoma, RCC, NSCLC, or other; *p* = 0.117), type of ICI (*p* = 0.714), or treatment line (*p* = 0.380). We separated the study cohort into three categories according to baseline NLR and fourth-week NLR change. The baseline characteristics of the three groups were similar, other than the LDH levels (Table 2).

### 3.2. Survival Analyses

During the 36.4 months of follow-up, 169 (73.2%) patients died, and 218 (92.2%) patients had any PFS event. The median OS and PFS of the cohort were 13.5 (95% CI = 10.10–16.90) and 4.98 (95% CI = 3.57–6.02), respectively. In univariate survival analyses, patients with higher NLR at baseline (<5 vs. ≥5, *p* = 0.013), 10% or over NLR increase in the fourth week of treatment (*p* = 0.002), higher ECOG performance score (0 vs. ≥1, *p* < 0.001), higher LDH levels (N vs. ≥ULN, *p* = 0.003), and higher CCI (<9 vs. ≥9, *p* = 0.002) had decreased OS. In contrast, the association between the combined use of chemotherapy (CT) or targeted therapy (TT) (*p* = 0.998) did not reach statistical significance. The PFS analyses were consistent with the OS analyses.

We conducted the multivariable survival analyses via a binary logistic regression model constructed by the statistically significant parameters in the univariate analyses. In multivariate analyses, a higher NLR at baseline (HR = 1.743, *p* = 0.002), 10% or over NLR increase in the fourth week of treatment (HR: 1.807, *p* = 0.001), higher ECOG performance score (HR: 1.552, *p* = 0.006), higher LDH levels (HR: 1.454, *p* = 0.017), and higher CCI (HR: 1.400, *p* = 0.041) were associated with decreased OS (Table 3). While there was a negative trend for lower PFS for all five parameters, the association reached statistical significance in patients with higher ECOG status (HR: 1.401, *p* = 0.017) and patients with an increase of 10% or higher at the fourth-week follow-up (HR: 1.544, *p* = 0.004) (Table 3). Additional sensitivity analyses for patient age, sex, and tumor type yielded consistent results.

### 3.3. Construction of the Prognostic Model

We incorporated the parameters with a statistically significant association with OS in the multivariable analyses to the prognostic survival model. We coded NLR-based parameters as 0-1-2 (0 = baseline NLR < 5 and fourth-week NLR increase <10%, 1 = baseline NLR ≥ 5 or fourth-week NLR increase ≥10%, and 2 = baseline NLR ≥ 5 and fourth-week NLR increase ≥10%) and other prognostic factors as 0 or 1 and calculated the prognostic score with the sum of individual parameters due to similar OS HRs for individual model parameters. The total score spanned from 0 to 5. We used the NLR2-CEL name for the scoring system based on an acronym of included parameters (baseline and on-treatment NLR, CCI, ECOG, and LDH).

**Figure 2:**
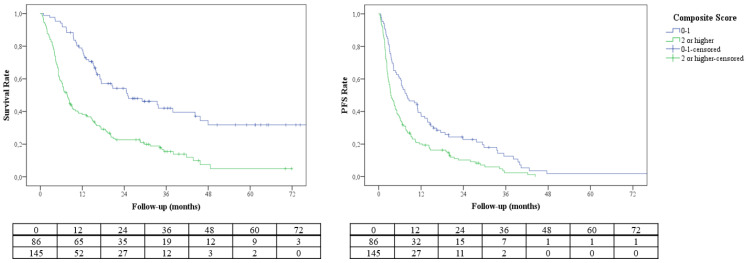
Kaplan–Meier analyses of overall survival and progression–free survival according to NLR2–CEL prognostic score (0–1 vs. 2 or higher).

**Figure 3:**
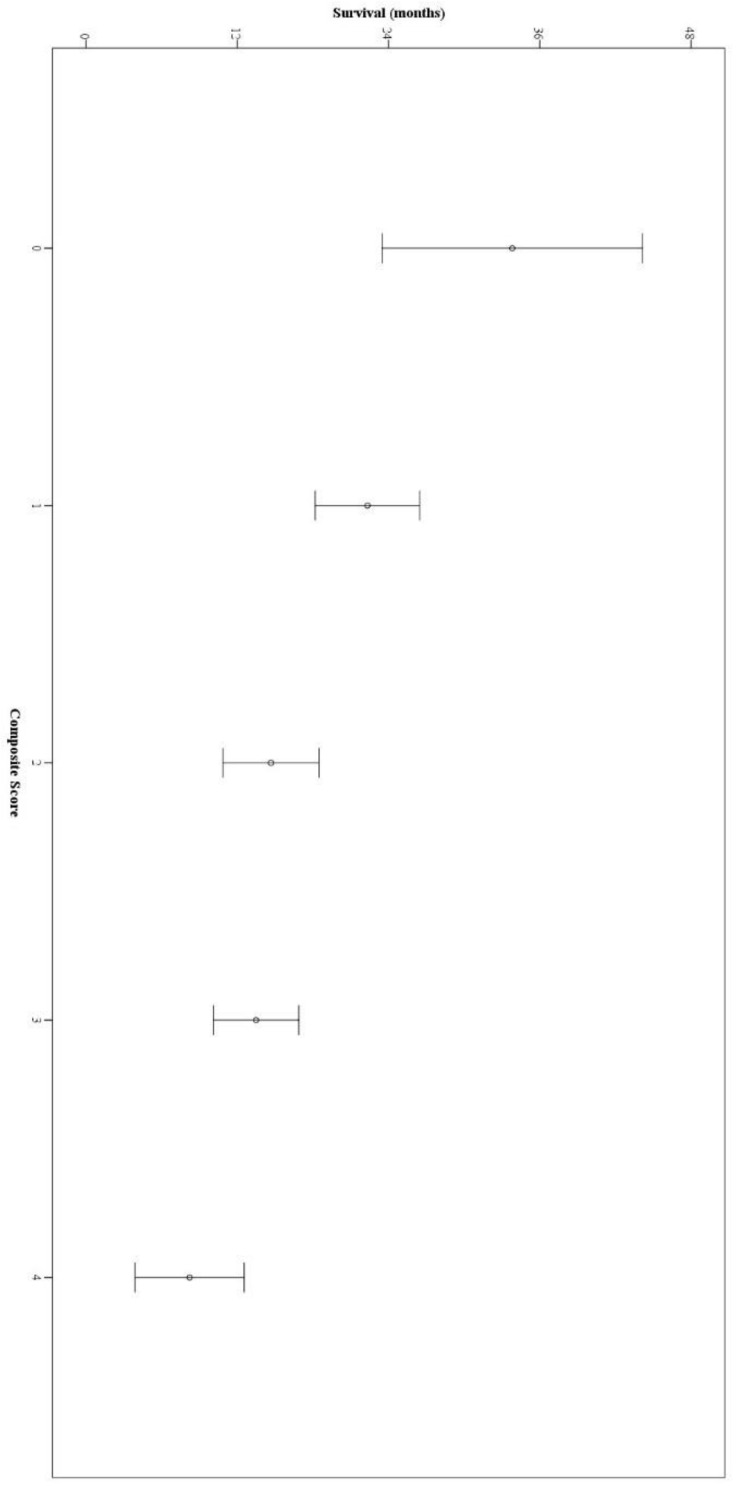
The association of the NLR2-CEL score with overall survival.

**Figure 4:**
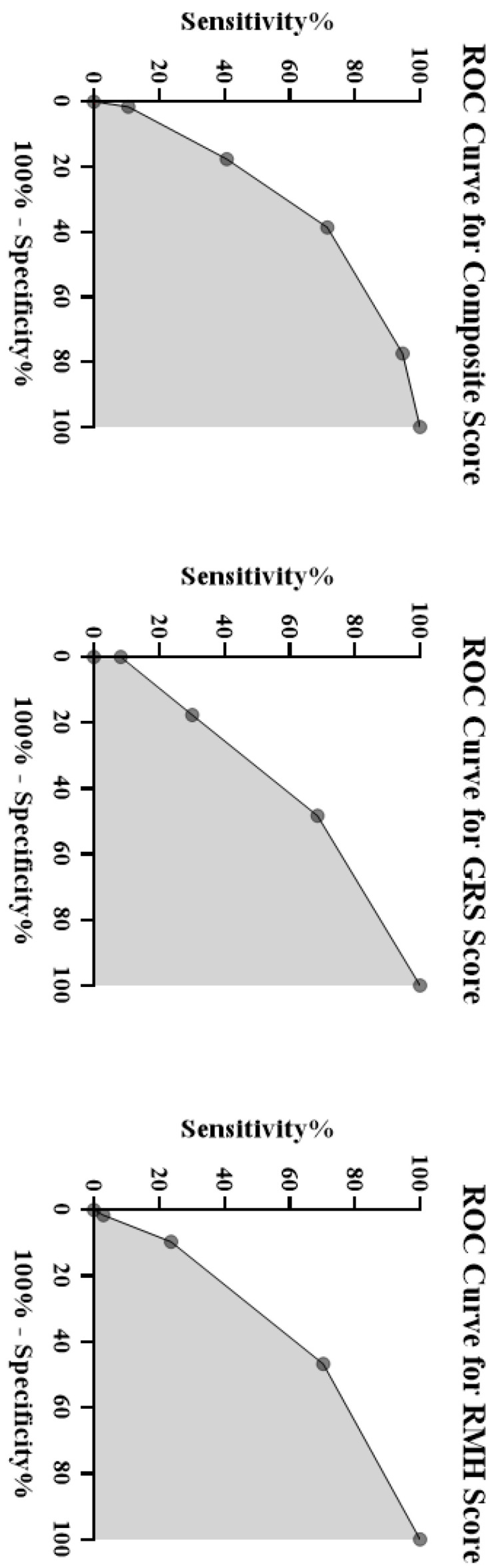
The receptor operating characteristics (ROCs) analyses of the NLR2-CEL score, GRS, and RMH scores for survival prediction.

The higher scores were associated with decreased OS and PFS in the Kaplan–Meier survival analyses (Figure 2). Compared to patients with the lowest scores, patients in the highest score group had significantly lower OS (HR = 7.967, 95% CI = 3.531–17.979, *p* < 0.001) and PFS (HR = 2.971, 95% CI = 1.570–5.620, *p* = 0.001). The prognostic score had a linear negative association with survival outcomes with lower OS with increased scores (Figure 3). The composite score had moderate success for OS prediction with AUC of 0.702 (95% CI = 0.626–0.779, *p* < 0.001). A score of 2 or higher had 71.6% sensitivity and 61.3% specificity for survival prediction. The AUC of NLR-based composite score had numerically higher AUC values than GRS (0.621, 95% CI = 0.541–0.701, *p* = 0.005) and RMH scores (0.639, 95% CI = 0.560–0.719, *p* = 0.001) for survival prediction (Figure 4).

## 4. Discussion

In this study, we evaluated the predictive performance of a compound score (NLR2-CEL) based on the baseline NLR and early NLR changes in two cohorts from different institutions. The increases in the compound score have a consistent and linear negative association with OS and PFS. The prediction power of the score was moderate and numerically non-inferior to two well-known prognostic scores, GRS and RMH.

The biomarkers for immunotherapy patient selection and prognosis prediction were not able to follow the speed and success of drug development and indication expansions [33]. Most candidate biomarkers involve tumor molecular characteristics and are subject to several limitations, including the one-dimensional nature, need for biopsy, and cost issues [34,35]. The compound prognostic scores based on simple clinical and laboratory parameters have recently gained significant interest for prognosis prediction in ICI-treated patients [36,37]. Similar to our study, most of these scores were developed in the basket immunotherapy cohorts and incorporated complete blood count and biochemistry parameters into the scoring systems; however, dynamic changes in the laboratory parameters are absent in these scores [30,32,38,39]. The most thoroughly investigated of these scores are the GRS and RMH scores [31,40,41]. Both scores include the baseline LDH (normal vs. ≥ULN) and albumin levels (3.5 g/dL vs. <3.5 g/dL), while the baseline NLR levels and number of metastatic sites were used in the GRS [30] and RMH scores [31], respectively. The predictive powers of these scoring systems were very variable in the reported studies and generally spanned between 0.60 and 0.90 [30,31,42,43,44].

The LDH levels were considered as a surrogate of tumor burden and tissue destruction, as well as tumor metabolism [45,46,47]. The prognostic role of LDH is well-defined in melanoma patients treated with ICIs, and the LDH levels are being used as stratification criteria in melanoma clinical trials [31,48,49,50]. Similarly, a worse prognosis was observed in ICI-treated patients with other indications, such as NSCLC and RCC in the observational studies [31,51,52,53,54]. The LDH levels were included in some of the prognostic models, albeit with different cutoffs (>ULN vs. >2× ULN) used for patient dichotomization [55]. We observed shorter OS and PFS in patients with higher LDH levels and used >ULN to define the higher LDH values due to relatively modest sample size and a small percentage of patients with LDH levels >2× ULN. 

In addition to LDH, NLR levels were among the most frequently investigated peripheral blood parameters in ICI-treated patients, and decreased OS was reported in patients with higher NLR levels in most studies; however, cutoff and population-related factors could affect the results [31,56,57,58]. In our analyses, the NLR values remained significant in the multivariable analyses and were included in the prognostic model. We used baseline NLR values as a prognostic parameter similar to previously published compound prognostic scores, such as the GRS and RMH score [30,31]. While the pretreatment values of these parameters are related to survival, changes in these markers could also aid in survival prediction [29,59]. However, in contrast to our study, most of these scoring systems did not include the dynamic changes in the peripheral blood cells in the equations [60,61]. Recent observational studies suggested that early changes of the peripheral blood markers could aid in prognosis prediction in-ICI treated patients with reflecting host–tumor interactions and host immune activation [62,63]. The response rates were better in patients with lymphocyte or eosinophil expansion under ICIs [31,64,65,66]. In contrast, a lower benefit with ICIs would be expected in patients with neutrophilic expansion due to protumorigenic and immunosuppressive properties of neutrophils secondary to the secretion of increased progranulopoietic cytokines and blunting of T-cell antitumor responses [67,68]. Considering this biological rationale, the changes in NLR values under treatment could benefit survival prediction. Li et al. reported better OS in patients with baseline and on treatment NLR of less than five in a large cohort of ICI-treated patients (*n* = 509) [29]. The median OS of patients with a moderate NLR decrease was 27.8 months, while the patients with a significant increase in NLR levels had a median OS of 5 months. We selected the percentage of NLR changes within the fourth week of treatment due to the receipt of two cycles of ICIs at that timeframe and data availability at this period. Additionally, we aimed to select a timeframe before the first radiological response evaluation. We observed lower OS and PFS in patients with a 10% increase from baseline at the fourth week of treatment in multivariable analyses and included this parameter in our prognostic score. We think that our observation supported the notion of adding early changes in peripheral blood markers to compound prognostic scores as a surrogate of changes in host immune status.

The ECOG performance status is a consistent predictor of survival in patients treated with chemotherapy or surgery and is a part of clinical oncology practice as a robust denominator of the patient’s general status and symptom burden [69,70]. While a significantly lower OS and PFS was observed in patients with an ECOG performance status of 2 compared to 0–1 [71,72], recent single-arm observational ICI studies reported significantly better survival in patients with 0 compared to patients with an ECOG performance status of 1 [73]. Additionally, ICI clinical trials primarily enrolled patients with ECOG status 0 to 1 only, and the ECOG status (0 vs. 1) was used as a stratification criterion in most clinical trials [74]. Based on the experience from clinical trials and a relatively low number of patients with an ECOG status of two or higher, we dichotomized patients as ECOG 0 vs. >0 in our model. We observed significantly lower OS in patients with an ECOG status of 1 or higher compared to ECOG 0 patients. 

In addition, different from the other models, we added CCI to a compound ICI-prognostic model. The CCI was developed and used to precisely quantify the comorbidity burden [75]. Additionally, CCI could be used as an indirect denominator of frailty in retrospective cohorts [76]. A large body of data demonstrated decreased survival and increased toxicity in patients with higher CCI treated with chemotherapy [77,78]. However, the role of CCI in the clinical management of ICI-treated patients is relatively unknown. In a recent publication from National Cancer Database, patients with higher comorbidity index had significantly decreased ICI use (HR, 0.85; 95% CI, 0.77–0.93) [79]. Another recent small study (*n* = 66) in non-small cell lung cancer patients treated with anti-PD1 treatment demonstrated decreased PFS and disease control rates in patients with CCI 7 or higher [80]. We used a different cutoff (CCI 9 or higher) for dichotomization and observed significantly lower OS in patients with higher CCI, while the PFS difference did not reach statistical significance. Based on our observations, we think that data CCI could be an adjunct to ECOG performance status for prognosis prediction and could be incorporated into the compound prognostic scores in ICI-treated patients.

The present study is subject to several limitations. First, the retrospective design and a heterogeneous patient group with a modest patient number in subgroups prevented us from conducting additional subgroup analyses; however, the sensitivity analyses according to tumor type yielded consistent results. Most of our patients were treated in the later lines and as ICI monotherapy, and that limited the generability of our results to patients treated in the countries with access to immunotherapy in the earlier lines and patients treated with ICI-based combinations. However, despite these limitations, we demonstrated the promise of an NLR-based compound score (NLR2-CEL) as a possible biomarker for prognosis prediction.

## 5. Conclusions

In conclusion, we observed a significantly lower OS in patients with poorer ECOG status, higher LDH levels, higher CCI, higher baseline NLR values, and increased NLR values under treatment. Our proposed prognostic score, NLR2-CEL, which encompassed these parameters, had a moderate predictive power for OS. If it could be validated in prospective cohorts, this compound prognostic scoring system could be used for prognosis prediction in ICI-treated patients.

## Figures and Tables

**Table 1:** Baseline patient characteristics of study population.

Clinical Feature	*n* (%)
Median Age (IQR)	61 (51–67)
Median CCI (IQR)	8 (7–9)
Sex	
Male	155 (67.1)
Female	76 (32.9)
ECOG PS	
0	132 (57.1)
1	69 (29.9)
2	26 (11.3)
3	4 (1.7)
Immunotherapy Agent	
Nivolumab	169 (73.2)
Atezolizumab	28 (12.1)
Pembrolizumab	20 (8.7)
Ipilimumab	13 (5.6)
Avelumab	1 (0.4)
Primary Tumor	
RCC *	49 (21.2)
Melanoma	49 (21.2)
NSCLC *	34 (14.7)
Other ^#^	99 (42.9)
Concomitant CT or TT ^+^	
Absent	176 (76.2)
Present	55 (23.8)
Line of Treatment	
1	31 (13.4)
2	91 (39.4)
3	48 (20.8)
4 or later	61 (26.4)

* RCC, renal cell carcinoma; NSCLC, non-small cell lung cancer; ^#^ Head and neck: 17, urothelial: 14, Hodgkin’s lymphoma: 11, small cell lung cancer: 10, other: 47; ^+^ CT, chemotherapy; TT, targeted therapy.

**Table 2:** The comparisons of baseline characteristics in the three groups according to baseline NLR and fourth-week NLR change.

		NLR < 5 and NLR < 10% Increase (*n* = 76)	NLR ≥ 5 or NLR ≥ 10% Increase (*n* = 138)	NLR ≥ 5 and NLR ≥ 10% Increase (*n* = 673)	*p*-Value
Age (median, IQR)		61 (54–66)	59 (50–67)	64 (60–70)	0.109
CCI (median, IQR)		8 (7–9)	8 (7–9)	8 (8–9)	0.290
Metastatic Site (median, IQR)		1 (1–2)	1 (1–2)	2 (1–2)	0.375
Primary Tumor	Melanoma	22 (28.9)	24 (17.4)	3 (17.6)	0.182
	RCC	15 (19.7)	27 (19.6)	7 (41.2)	
	NSCLC	9 (11.8)	23 (16.7)	2 (11.8)	
	Other	30 (39.5)	64 (46.4)	5 (29.4)	
LDH	Normal	52 (68.4)	68 (49.3)	9 (52.9)	0.025
	>ULN	24 (31.6)	70 (50.7)	8 (47.1)	
Charlson Comorbidity Index	<9	52 (68.4)	91 (65.9)	9 (52.9)	0.477
	9 or higher	24 (31.6)	47 (34.1)	8 (47.1)	
Concomitant CT or TT	Absent	63 (82.9)	100 (72.5)	13 (76.5)	0.230
	Present	13 (17.1)	38 (27.5)	4 (23.5)	
Baseline Liver Metastasis	Absent	50 (65.8)	96 (69.6)	12 (70.6)	0.834
	Present	26 (34.2)	42 (30.4)	5 (29.4)	
ECOG	0	46 (60.5)	74 (53.6)	12 (70.6)	0.315
	1 or higher	30 (39.5)	64 (46.4)	1355 (29.4)	
ORR	Absent	41 (57.7)	89 (70.6)	12 (85.7)	0.057
	Present	30 (42.3)	37 (29.4)	2 (14.3)	

**Table 3:** Cox regression analysis of the NLR-based compound prognostic score and overall survival and disease-free survival.

	Progression-Free Survival			Overall Survival		
	Hazard Ratio	95% CI *	*p*-Value	Hazard Ratio	95% CI *	*p*-Value
CCI (<9 vs. ≥9)	1.193	0.890–1.600	0.238	1.400	1.014–1.932	0.041
Baseline NLR (<5 vs. ≥5)	1.354	0.997–1.839	0.053	1.743	1.227–2.476	0.002
Fourth-week NLR increase (<10% vs. ≥10%)	1.544	1.152–2.068	0.004	1.807	1.294–2.524	0.001
ECOG (0 vs. ≥1)	1.401	1.061–1.848	0.017	1.552	1.134–2.123	0.006
LDH (N vs. ≥ULN)	1.219	0.926–1.605	0.158	1.454	1.069–1.976	0.017

* 95% CI: 95% confidence interval.

## Data Availability

The data used to support the findings of this study are available from the corresponding author upon request.
